# Validation of an Automated Cough Detection Algorithm for Tracking Recovery of Pulmonary Tuberculosis Patients

**DOI:** 10.1371/journal.pone.0046229

**Published:** 2012-10-10

**Authors:** Sandra Larson, Germán Comina, Robert H. Gilman, Brian H. Tracey, Marjory Bravard, José W. López

**Affiliations:** 1 College of Osteopathic Medicine, Michigan State University, East Lansing, Michigan, United States of America; 2 Laboratorio de Ingeniería Física, Facultad de Ciencias, Universidad Nacional de Ingeniería, Rimac, Lima, Perú; 3 Department of International Health, Johns Hopkins Bloomberg School of Public Health, Baltimore, Maryland, United States of America; 4 Department of Electrical and Computer Engineering, Tufts University, Medford, Massachusetts, United States of America; 5 Department of Internal Medicine, Massachusetts General Hospital, Boston, Massachusetts, United States of America; 6 Unidad de Epidemiología, Hospital Nacional Dos de Mayo, Lima, Perú; McGill University, Canada

## Abstract

**Background:**

A laboratory-free test for assessing recovery from pulmonary tuberculosis (TB) would be extremely beneficial in regions of the world where laboratory facilities are lacking. Our hypothesis is that analysis of cough sound recordings may provide such a test. In the current paper, we present validation of a cough analysis tool.

**Methodology/Principal Findings:**

Cough data was collected from a cohort of TB patients in Lima, Peru and 25.5 hours of recordings were manually annotated by clinical staff. Analysis software was developed and validated by comparison to manual scoring. Because many patients cough in bursts, coughing was characterized in terms of *cough epochs*. Our software correctly detects 75.5% of cough episodes with a specificity of 99.6% (comparable to past results using the same definition) and a median false positive rate of 4 false positives/hour, due to the noisy, real-world nature of our dataset. We then manually review detected coughs to eliminate false positives, in effect using the algorithm as a pre-screening tool that reduces reviewing time to roughly 5% of the recording length. This cough analysis approach provides a foundation to support larger-scale studies of coughing rates over time for TB patients undergoing treatment.

## Introduction

Tuberculosis (TB) is among the world's most deadly infectious diseases despite the long-standing availability of effective treatment. The steady emergence of multi-drug resistant (MDR) and extremely drug-resistant (XDR) forms of TB is a cause of concern. Globally MDR TB accounts for roughly 3.6% of all TB cases, but accounts for up to 28% of TB cases in some regions [1]. The emergence of MDR and XDR TB is very worrying due to the increased difficulty of treating these forms of tuberculosis.

The gold standard for TB diagnosis is laboratory culture and analysis. However, many high-incidence TB regions are in the developing world, where access to skilled laboratories and culture-based methods is lacking. Therefore, diagnosis of pulmonary TB is made exclusively by sputum smear or clinical suspicion alone due to lack of laboratory access. Following the WHO TB program, patients start first-line therapy, and treatment failure is only recognized 4–6 months later. Treatment failure may be due to MDR TB, lack of patient compliance, or other reasons. Patients who fail treatment have increased risks of morbidity and mortality, and also continue to be infectious, spreading disease to others.

Clinicians without access to laboratory culture would benefit greatly from lab-free early detection methods to identify patients who are failing treatment. Recent developments in fast DNA-based screening are promising, but still under development [2]. Our long-term goal is to evaluate whether cough analysis could provide a low-cost means for detecting treatment failure. This approach builds on a study that found cough rates (counts/hour) drop by roughly 50% in the first two weeks of treatment for patients who are responding to treatment [3]. The infrastructure needed for an automated cough monitoring system is relatively simple and low-cost (portable recording units and access to computers, either locally or via telecommunications).

Our work leverages progress in low-cost consumer electronics which allows ambulatory systems that can record for extended time periods [4], [5]. We also leverage previous algorithmic work in automated cough counting [6]–[11]. Fully automated analysis is key for protecting patient privacy, but is difficult as patient recordings may include very large amounts of environmental noise. Our dataset is fairly challenging and extraneous noises (speech, traffic, bangs, etc.) are common. Our recordings are made in a mix of hospital and home environments. Patients showing signs of recovery after one week of treatment were sent home with a recorder and recorded at home while treatment continued, until the data collection protocol was complete.

This paper reports our results with a pilot data collection and development of a cough detection algorithm. In this initial phase, we seek to develop an analysis approach that is sufficiently accurate to evaluate the clinical utility of cough analysis for TB patients. Our longer-term goal is development of a fully automated cough analysis system.

## Results

**Figure 1:**
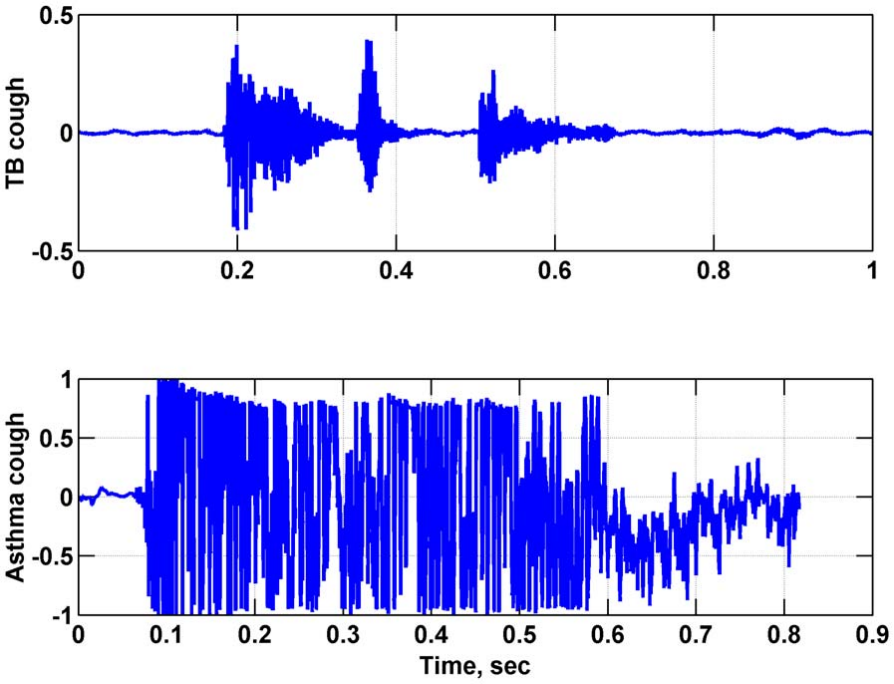
Waveforms of a double-cough from a TB patient (upper) and of an asthma patient (lower).

The validation dataset, consisting of 49 mp3 audio files each of 30 minutes duration, was analyzed using the algorithm and also reviewed manually by two nurses and a specialist. The cough waveforms reviewed differ depending on the patient and pathologies, both in terms of pitch, amplitude and frequencies. Figure 1 shows example coughs from TB and asthma patients. Note that these waveforms both show one *cough episode* consisting of several closely spaced cough events.

Within the cough analysis literature, there are several metrics used to quantify cough, and to quantify algorithm performance. A basic metric for quantifying cough is coughs/hour (also called *cough count*). As discussed by Smith [4] one can also quantify cough in terms of cough *bouts* or *episodes*, where an episode is defined as one or more cough events that are closely spaced in time. In [5], a cough *epoch* was been defined as a series of coughs in which the end of one event is separated from the beginning of the next event by less than 2 seconds. Smith also proposed the *cough-second* metric, or the number of 1-second intervals per hour that contain a cough. As stated in [6], “there is little to commend any particular method of quantifying cough over any other”.

During manual review, individual cough events were identified. We noted that in some cases, the algorithm tended to unite closely timed individual cough events (as in Figure 1, and File S1) into a single detected cough. Because many patients cough in bursts, we found low sensitivity in detecting individual coughs (51.4%, as calculated using the Data Analysis and Statistical Software (STATA®).

While the algorithm could in principle be re-tuned to separate these events, the literature indicates that counts of cough epochs appear to be as clinically meaningful as counts of individual coughs [4], [6]. Thus we merged groups of individual coughs into cough epochs, following two definitions. In the first definition, (denoted *epoch1*) we grouped coughs whose start times were within 2 seconds into the same epoch. Under the second definition (*epoch2*) we followed the definition in [5], i.e. we grouped coughs together if the gap between the end of one event and the start of the next is <2 sec. While both epoch types can be calculated from algorithm output (and are compared below), only the event start times were noted during manual review. Thus, manual epochs can only be calculated using the *epoch1* definition.

The start times of epochs formed from manual review and algorithm outputs under the *epoch1* definition were compared. If these times matched within +/−0.2 sec, we declared that the algorithm had successfully found a cough epoch, and increased the number of true positives (TP). Epochs missed by the algorithm constitute false negatives (FN), while epochs found only by the algorithm constitute false positives (FP). We therefore calculate algorithm sensitivity as TP/(TP+FN), and report a rate of false positives/hour as FP/(# hours of data analyzed). Our algorithm sensitivity in reporting epochs was 75.5%.

The false positive rate is often captured in terms of specificity (equal to TN/(TN+FP), where TN is the number of true negative results). Within the cough literature, there are several approaches to calculating TN. Matsos *et al.* [9] proposed a two-stage process, in which they first detect acoustic events, then classify those events into cough or non-cough. They then report a classification stage specificity, i.e. they sort their *detected* events into TP, FP, TN and FN. This metric, often known as “Birring specificity”, is useful in comparing different classification approaches. However, as pointed out by Vizel *et al.* [11] this definition does not reflect performance of the overall system, which is of interest to a clinical end user. Vizel *et al* therefore calculate TN from the number of 1-second intervals during which no cough was detected by either manual or automated means.

Following Vizel *et al* [11], we defined specificity by first finding all 1-second intervals during which no coughs were found during manual review, then partitioning these into true negatives and false positives. Under this definition our algorithm specificity was 99.6%, which actually exceeded the 96% value reported in [11] (although [11] examined a very different dataset, and reported >90% sensitivity). Our “Birring specificity” was 87%. We also calculated the number of false positives per hour, and found that we had a median false positive rate of 4/hour (lower quartile of 0/hour, upper quartile of 8.5/hour), which is higher than the median 1.2 false positives/hour calculated from results in [11] or the median 0.8 false positives/hour reported by [10].

To eliminate false positives, we implemented a simple user interface for manually reviewing detected coughs. This review required approximately 0.63 minutes per hour of audio recording, representing a speedup of roughly 96-fold as compared to reviewing the full recording at normal playback speed. This speedup compares favorably to the semi-automated HACC system [8], for which ∼1.5 minutes was reported for reviewing one hour or recording. Use of this system in this way, as a pre-screening step, eliminates false positives while not affecting sensitivity. After review, sensitivity remains 75.5%, our “Birring specificity” is 99.3%, our 1-sec interval specificity (following Vizel) was 99.9, our median false positive rate was 0/hour, our mean false positive rate was 0.5/hour, and our mean true positive rate was 6.8/hour. We refer to these results as ‘semi-automated’.

**Figure 2:**
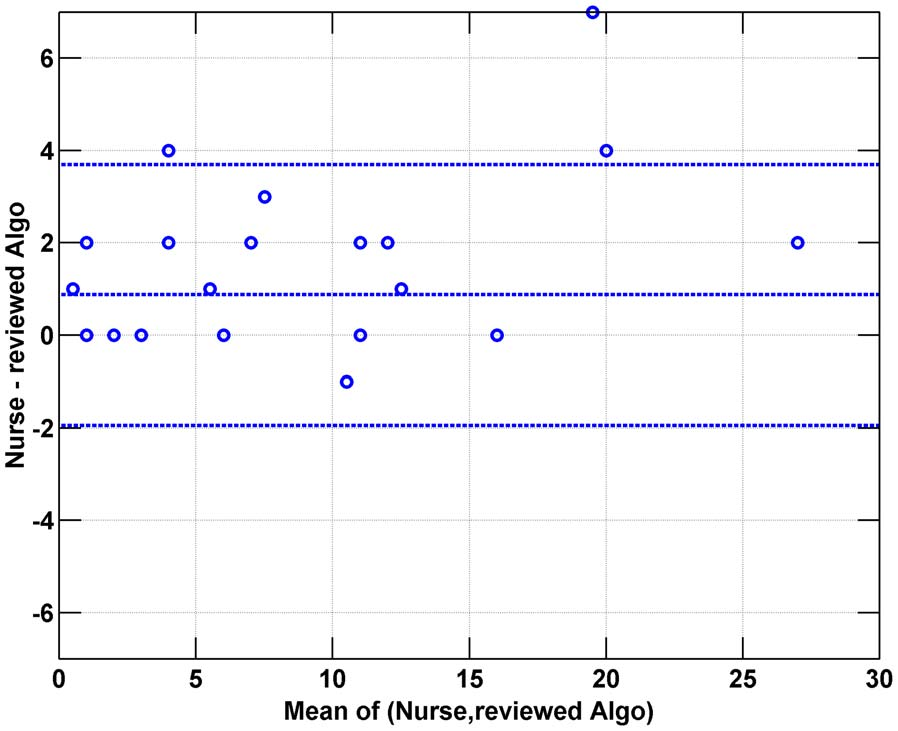
Bland-Altman plot comparing the number of epochs (definition *epoch1*) found by the nurses and the reviewed algorithm (i.e. semi-automated approach). The mean of the two estimates (used in place of a gold standard) is plotted vs. the difference between nurse and semi-automated results. The mean bias and limits of agreement (+/−1.96 σ) are also shown. The plot shows the bias is not statistically significant and there is no evidence of changing agreement as a function of cough epoch count.

Semi-automated output and manual results are compared in a Bland-Altman plot in Figure 2. For each 30 minute recording, the mean of the manual and semi-automated epoch counts are compared to the difference. A bias of 0.9 epochs is seen, corresponding to the fact that algorithm detection sensitivity is <100%. The bias is not statistically significant (limits of agreement are −2 to 3.7 epochs/hour), and there is no evidence that the performance depends on the underlying cough rate. A Bland-Altman analysis for the fully automated result (i.e., no review) shows much larger spread (limits of agreement −22 to 17 epochs).

**Figure 3:**
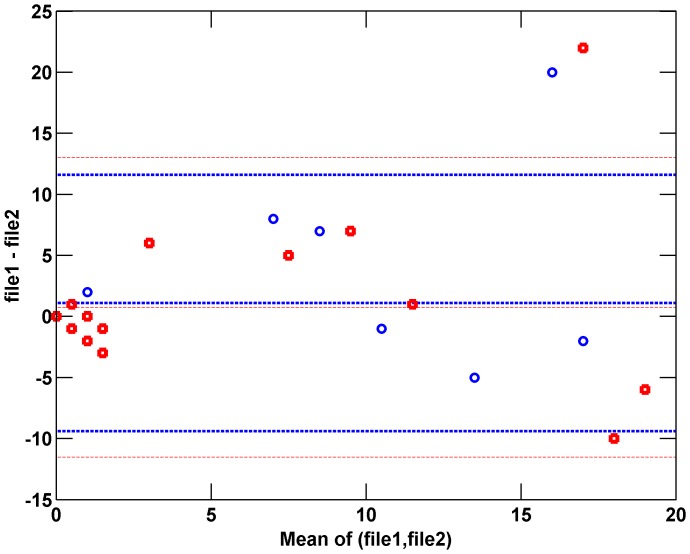
Bland-Altman plot comparing repeatability of semi-automated results (blue circles) to repeatability of nurse findings (red squares). In this comparison, the first and second files for each day (‘file1’ and ‘file2’) were compared. Repeatability is similar for nurse assignments and semi-automated algorithm results.

By design, the validation dataset included two data files per day for each subject to allow repeatability to be explored. We examined the repeatability of epoch counts, for the semi-automated approach as compared to the nurses, by identifying pairs of files for which results where available (both files were recognized by the algorithm as having good quality data). Figure 3 shows a Bland-Altman plot comparing repeatability of both the algorithm and nurses in determining cough epochs. There is little bias and the limits of agreement and data scatter are comparable for both nurse and semi-automated algorithm repeatability. This suggests the repeatability of manual and semi-automated analysis is similar. When re-analyzing a single file, the algorithm repeatability is 100%.

**Figure 4:**
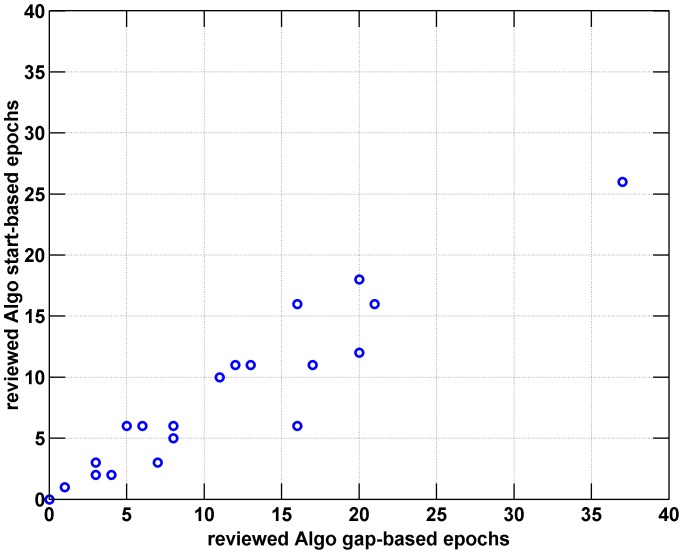
Scatterplots showing the number of epochs found under ‘*epoch1* ’ and ‘*epoch2*’ definitions, for semi-automated algorithm results. Note that the ‘*epoch2*’ definition cannot be applied to nurse assignments in our dataset. The correlation coefficient between the two definitions is 0.97.

Figure 4 compares the number of epoch calculated under the *epoch1* and *epoch2*, for semi-automated outputs. As expected, the *epoch1* definition is more restrictive and therefore results in fewer epochs being found. However, correlation of the measures is high (Spearman correlation coefficient >0.97) suggesting that our validation using the *epoch1* definition suggests similar results for the *epoch2* definition.

**Figure 5:**
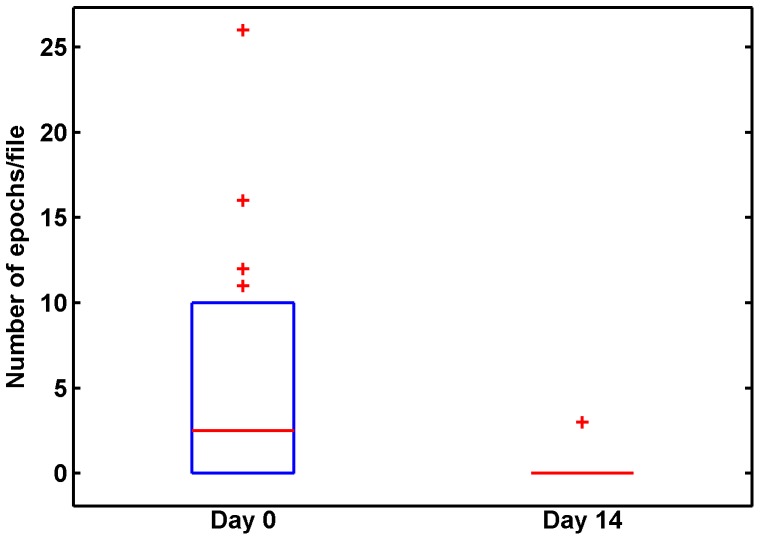
Boxplot comparing semi-automated estimate of cough count at day 0 and day 14. The plot shows 25^th^, 50^th^, and 75^th^ percentiles, with outliers (1.5*IQR) are shown as ‘+’. At Day 14, the box collapses as 25^th^, 50^th^, and 75^th^ percentiles are all zero.

Finally, we compared results for treatment day 0 (baseline) vs. day 14. In total on day 14, the nurses detected 10 cough epochs, 6 of which were detected by the algorithm, giving a sensitivity of 0.6. However, the four undetected epochs were spread across four different recordings. Figure 5 shows a boxplot of the number of cough epochs per file, for all drug-susceptible patients at treatment day 0 (baseline) vs. day 14 (because we have only one MDR patient in the study, it is not meaningful to examine MDR statistics). While the sample size for the validation study is small, there is a clear drop in cough. Post-treatment, the 25^th^, median, and 75^th^ percentiles for cough epochs are all 0; two patients were outliers with 3 coughs/file. The Kolmogorov-Smirnov two-sample test rejected the hypothesis that the pre- and post-treatment cough epochs are drawn from the same population (p<0.02), i.e. it indicates that the distributions are different.

## Discussion

We have developed a cough analysis system that builds on previous approaches [8]–[10] and have applied it to a cohort of TB patients. In developing a cough analysis system, our near-term goal is to develop an automated tool for characterizing changes in cough events/hour for patients being treated for tuberculosis. This will allow us to test our hypothesis that cough analysis can provide a laboratory-free tool for tracking patient recovery.

A challenging (and realistic) aspect of our dataset is that patients are wearing recording systems while going about their daily activities (note that in Peru, TB patients are not routinely hospitalized). Our recordings include extensive recordings of speech as well as traffic and construction noise, barking dogs, children playing musical instruments, etc. Thus the number of recorded acoustic events exceeds the number of coughs by orders of magnitude, meaning that the analysis algorithm must be extremely specific to avoid high false alarm rates. Within the current study, this led us to conclude that the algorithm should be used in a semi-automated manner, essentially as a prescreening step to identify candidate coughs. While this solution is clearly not optimal, the speedup obtained is significant enough that it provides a manageable path to our initial goal, which is quantifying clinical cough changes in subjects enrolled in our pilot study. Encouragingly, Figure 5 shows a marked decrease in cough after treatment within our validation study data, though of course a much larger data study is needed.

We note that many published results [8]–[10] are also semi-automated (note that [10] includes a learning phase that helps the algorithm improve as manual review is done, but roughly 1/3 of detected events were still manually reviewed). Our sensitivity of 75.5% is similar to other semi-automated approaches ([8] reported 80% cough sensitivity, while [9] reported 71–82% depending on settings). Fully automated systems we are aware of include [11] as well as the VitaloJAK and Lifeshirt systems [4] but these systems are much more expensive, exploiting multiple sensors.

Prior to prescreening, our algorithm had a significant number of false positives. We strongly suspect that our dataset was noisier with less well controlled recordings than some other datasets reported in the literature, as microphone placement was done by patients, not healthcare workers. However, this is difficult to verify objectively (although sample audio clips are available with this article). While our training data contained a reasonable diversity of cough and non-cough noises, it has long been recognized that larger training sets are beneficial in speech processing [12], so gains could be likely realized given a larger training set.

Several of the algorithmic approaches used here may be of use to other cough analysis efforts. In our initial work, we implemented an algorithm combining methods used in previous semi-automated approaches [8]–[10]. While performance was good in quiet recordings, we observed high false positive rates in other recordings. As a result, we developed two modifications that allowed us to improve performance. First, we developed event detection logic that only triggers on events exhibiting the time evolution expected for cough, i.e. a rapid increase in acoustic energy relative to the noise floor. Second, we developed logic for automatically flagging sections of the recording during which there are technical problems, or during which the background noise is rapidly varying, indicating an extremely noisy acoustic environment or technical recording problems. Audio examples are provided along with the article. These flagged data sections could be manually reviewed, though they are infrequent enough that we have instead chosen to discard them from analysis. Importantly, these data sections are flagged automatically without need for manual input. We have a large clinical dataset consisting of continuous 24-hour recordings over multiple days for each patient. We therefore anticipate being able to assess changes in cough during recovery even if some noisy portions are not analyzed.

In addition, we describe a ‘divide-and-conquer’ clustering algorithm that identifies the most informative data examples for machine learning algorithms. This approach was necessary to reduce memory requirements for classifier training to a manageable size. It may prove useful for applications where the classifier is updated as new data become available, as it only requires storing a single representative of each previously identified cluster.

## Materials and Methods

Here we describe the methods used in our study, focusing on two key areas: data collection and manual review, and the algorithm used for automated analysis. An earlier version of the algorithm is described in [13]. Here we focus our algorithm discussion on new data quality metrics as well as giving a more detailed discussion of novel parts of the algorithm.

### Ethics statement

This study received IRB approval from Hospital Nacional Dos de Mayo, Associacion Benéfica Prisma (Lima, Peru), and Johns Hopkins University.

### Data collection and manual review

The data collection was conducted at Hospital Nacional Dos de Mayo in Lima, Peru. This public national tertiary referral hospital also operates as a community hospital for the surrounding inner-city area.

We collected a large dataset of patients for our study. Exclusion criteria included pregnancy, previous TB treatment, history of taking medication within the last month, and age less than 18 years old. From this dataset, fifteen patients were randomly selected who had full 24-hour acoustic recordings corresponding to two specific days of treatment in the pilot study (as a note, [14] also used 15 subjects). Of these patients, 4 were female, 4 were HIV positive, and one was multi-drug resistant. Median age of the 15 patients was 33 (range 19–47). Recordings were obtained using a Marantz PMD 620 handheld recorder and an Audio-Technica AT899 sub-mini microphone attached at the patient's lapel.

From each patient's data, two 30-minute subsets on day 0 (start of treatment) and on day 14 (well into treatment) were randomly selected, yielding a total of 60 30-minute cough recordings. Each recording was screened to ensure that sounds were recorded (that the recording was not merely silent), resulting in elimination of one recording, leaving 59 files. By choosing data from day 0 and day 14, we sought to capture performance at different times during treatment.

Because our recordings were ambulatory and often contained significant environmental noise, the remaining 59 recordings were screened for quality by our analysis algorithm using automated methods described below. Of these recordings, ten were automatically flagged as having data quality issues (two with recording levels too low, and eight with noise levels too high). Thus the working dataset consisted of 49 recordings.

#### Manual review

Two nurses independently reviewed these dataset files using Audacity software [15] to record the start time of every cough event during each recording. This was repeated three months later, giving a total of four reviews of each recording. The coughs were tabulated by the nurses in a spreadsheet. The nurses generally ranged within 0.2 seconds of each other in detecting cough events. Discordant events were resolved by the two nurses and one of the authors (SL) who came to consensus about the event's status as a cough or non-cough. Discordant events were relatively few (19 of 1493 events) and the kappa statistic measuring agreement between nurses was 0.85. The average cough time of the four time values for each event (the two independent nurses at two different times) was computed and this value was considered as the “true” time for each cough event.

### Algorithm description

**Figure 6:**
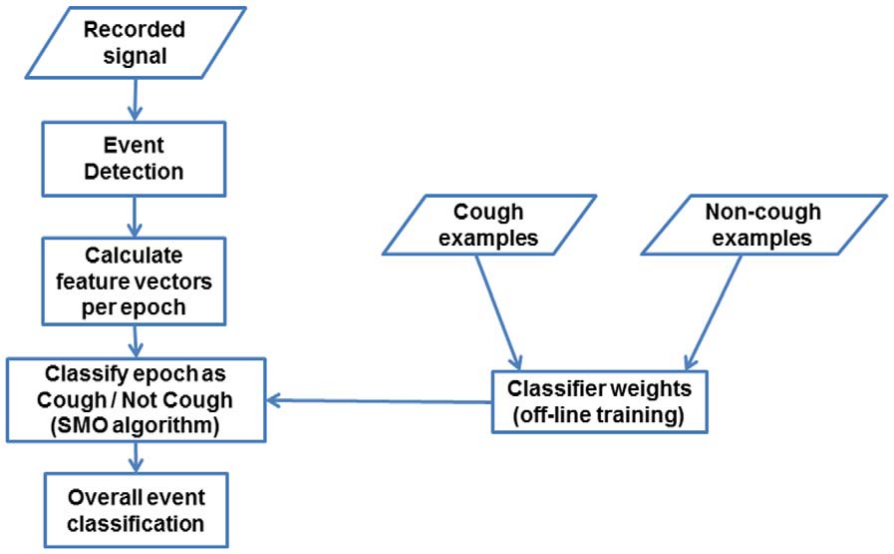
High-level flowchart of cough detection algorithm.

Our overall algorithm flow is shown in Figure 6. Like previous approaches [8]–[11], processing is divided into an *event detection* stage, followed by an *event classification* stage. As described in detail below, we initially detected events using a simple energy detector, but found better results with an approach that seeks events whose onset shows the rapid energy increase typical of cough.

An earlier version of our algorithm is described in [13]. An important algorithmic concept added since that publication is the concept of *data quality flags*. Our dataset contains a minority of recordings in which there are either significant technical problems (typically, extremely low signal level due to microphone problems) or extremely high variable levels of background noise. By adding automated flags to detect these conditions, we can either trigger a manual review or discard the data files (as was done for ten files as described above).

To aid in event classification, time-frequency analysis is used to capture the acoustic characteristics of the detected event. Events are broken up into 50% overlapped frames of 32 ms length, and are analyzed by computing the Mel frequency cepstral coefficients (MFCC), defined as:
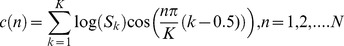
(1)Here, *N* is the number of coefficients to be calculated, and *Sk* are the outputs of *K* different filterbanks, found by weighted sums of the short-time Fourier transform magnitude over a set of frequency bands with center frequencies chosen to approximate the human auditory system response [16]. First and second time derivatives of the MFCC coefficients are then numerically calculated, and the MFCC coefficients and their time derivatives are collected into a feature vector:
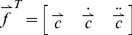
(2)While these features were originally developed for speech analysis, they have also been proven to be useful for cough sounds analysis [8]–[10]. These features for individual frames are fed into a classifier (trained offline) to determine whether or not the frame has the characteristics of cough. Finally, the decisions for individual frames are combined to arrive at an overall decision for the event.

#### Event detection

Our first implementation of a cough analysis algorithm detected acoustic events using a simple energy detector, following previous work [8]–[10]. In this approach, a signal envelope was estimated by squaring the input signal and smoothing it with a centered moving average filter (a rectangular filter of length 0.05 s). The detector then identified events as regions of time where the signal exceeded an energy threshold. Events that were separated by a very small gap in time (0.1 s) were merged together into one event (similar to a morphological closing operation in image processing).

**Figure 7:**
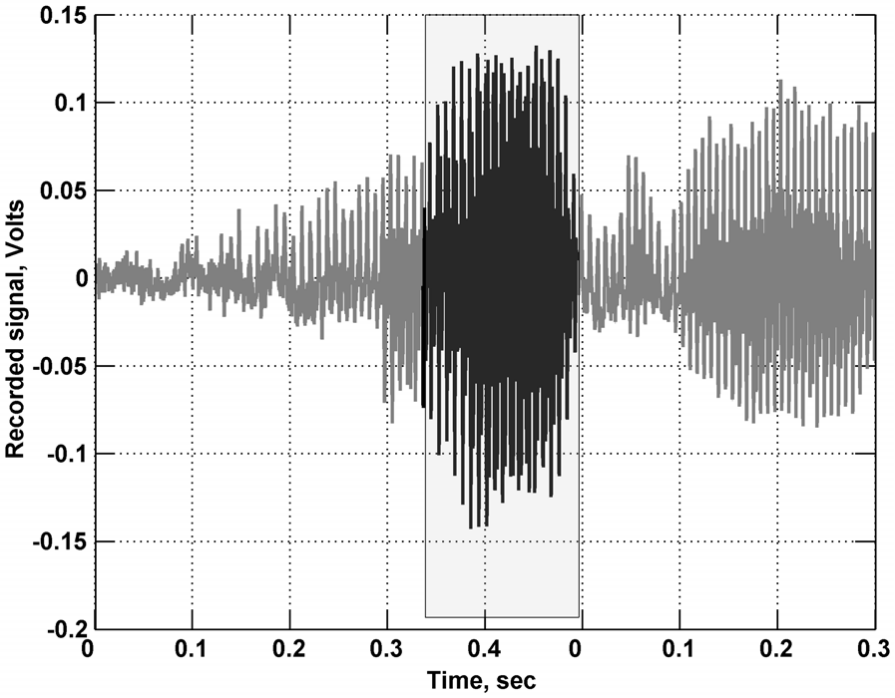
Example issue with simple energy detector. The threshold may miss the start of the acoustic event and is frequently crossed during speech events (shown above), increasing the chance of misclassification.

We found several difficulties with this approach, illustrated in Figure 7. In some cases the energy threshold was not crossed until partway through the event. This is undesirable as the initial portion of the event is important for cough classification, as the initial explosive phase is highly characteristic of cough. More commonly, the energy detector triggered on speech signals, as shown in the Figure. Speech signals may have significant energy, but typically show a more gradual change in signal energy than coughs. These detected speech events were then passed through the second event classification stage. Although the large majority of these speech events were correctly rejected, the overall effect was to increase the number of false positives. We note that in most of our recordings, speech events outnumber cough events by 1–2 orders of magnitude, so even a small rate of misclassification can have significant consequences for overall performance. A final difficulty (not shown in the figure) is that the background noise levels differ greatly for various recordings in our dataset, so use of a fixed noise threshold is problematic.

**Figure 8:**
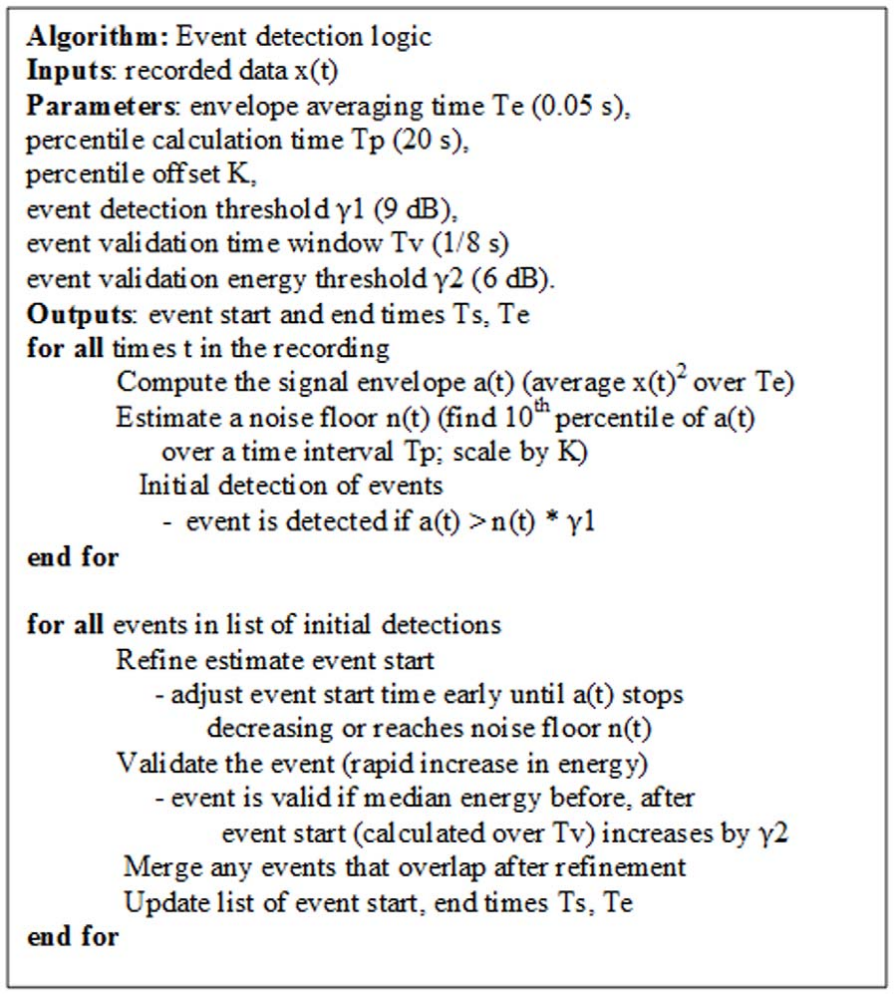
Pseudo-code for event detection logic.

We therefore developed an improved detection scheme which searches for the rapid increase in signal energy that is characteristic of cough. To account for the variety of noise environments in our dataset, we make use of a time-varying estimate of the noise background. Pseudo-code for the detection algorithm is shown in Figure 8. The parameters shown were determined empirically. To reduce computation, percentiles were calculated for a subset of windows with 75% overlap and then interpolated to each time sample.

#### Event classification: off-line training

A subset of the recorded data was used as training data. Because of data availability at the time training was done, the training data is mainly taken from two male subjects. However, the training data included a wide diversity of non-cough events, and we actually saw slightly better sensitivity with female patients (81%). A graphical user interface was built to support classifier training. Recordings were processed to identify events, which were then played for the user. To create a large library of both cough and non-cough events, the simple energy threshold detector described above was used, with a low threshold to allow sensitive detection of events. Events were manually reviewed classified as ‘cough’, ‘not cough’, or ‘unclear’. ‘Unclear’ events were either ambiguous in nature or were coughs with other sounds in the background, such that they were judged unsuitable for classifier training. This process yielded 418 cough events, 1980 ‘not cough’ events, and 75 ‘unclear’ events.

Once each event was manually labeled, it was split into frames and the feature vector shown in Eq. 2 was calculated. All frames within the event were labeled as ‘cough’ or ‘noncough’ based on the manual classification of the overall event. This yielded a total of 13,429 cough frames and 43,925 non-cough frames for training. There is a possibility for misclassification in the training data, as not every frame in a ‘cough’ event may contain cough sounds. However, the large number of example frames should help mitigate this problem.

**Figure 9:**
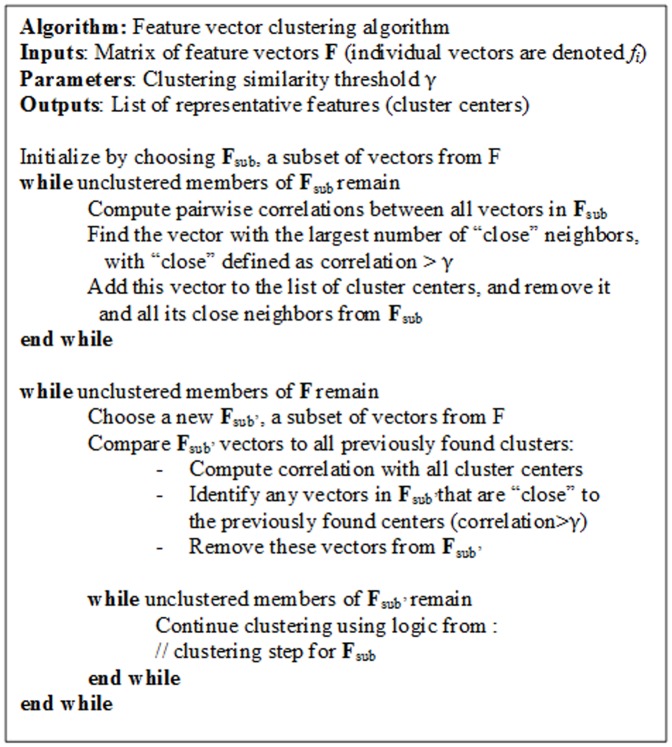
Pseudo-code for clustering algorithm.

Classifier training was performed using the Weka 3.6 software [17] which allows testing of a wide range of different machine learning algorithms. Training using the full dataset of over 57,000 frames is computationally infeasible, and also of limited value as many frames are very similar. Thus, we applied a previously developed ‘divide and conquer’ clustering method suitable for large datasets [18]. During the algorithm, representative ‘cluster centers’, or exemplar frames of different types of sounds, are computed. New frames are associated with these centers if they have correlation >0.95, i.e. if
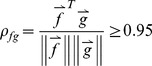
(3)where *f* and *g* are feature vectors of the form shown above, and ∥ ∥ denotes the 2-norm of the vector. Pseudo-code for the clustering algorithm is shown in Figure 9. For our training data, this clustering approach yielded 2074 vectors, representing half cough events and half non-cough.

Using 10-fold cross-validation, we compared performance of neural networks, support vector machines (SVM), and sequential minimal optimization (SMO) algorithms. Details of the comparison are in [13]. Performance of the various methods was similar, so we chose the SMO approach for ease of implementation.

#### On-line event classification

During on-line processing, the SMO classifier trained as described above was used to classify individual frames with each detected event. In a final step, the decisions for individual frames within each event are combined to classify the overall event. A previously proposed approach [8] is to average classifier scores from all frames within an event. We found improved performance by first identifying the 1/3 of contiguous frames that have the most ‘cough-like’ scores, then averaging the classifier outputs for those frames. This approach helped avoid misclassification when the detected event contained a mix of cough and other vocalizations.

#### Data quality flags

While an ideal cough detection algorithm would rival the abilities of a human listener, certain types of files have the potential to cause significant problems for automated analysis. A final analysis stage is therefore the calculation of data quality flags. Our software outputs one of three flags, defined as follows:

- Type 0: normal file
- Type 1: low-amplitude file (technical problem with recorder); maximum value in file<threshold (threshold is 30% of full dynamic range; note that typical files use full dynamic range)
- Type 2: high-noise file; variation (max-min) in estimated noise floor during the file exceeds a threshold. Triggered by very noisy environments or by technical problems (intermittent electrical connections)

Thresholds for these tests were found through engineering judgment. Files S2, S3, S4 contain example audio clips of all three types of files.

While algorithmically very simple, the concept of flagging difficult data cases proved very helpful practically. Because much of our dataset was collected in daily-life settings, microphone placement and other important determinants of data quality depend on the patients wearing the microphone, rather than on specially trained clinical staff. Low amplitude Type 1 files constitute roughly 4% of our dataset. In these files, the algorithm detects few acoustic events, increasing the false negative (FN) rate. High noise Type 2 files constitute roughly 17% of our dataset. In these files, our event detection logic (which relies on a smooth noise estimate) does not perform well, increasing the rates of both false positives and false negatives. As noted above, the data quality flag can either trigger manual review or discarding of the file from analysis results.

## Supporting Information

File S1 **This file contains an example of a double-cough from a TB patient.** Several patients (such as this one) nearly always cough in a characteristic double-cough pattern.
(WAV)

File S2 **This file is an example of a file that was rejected due to very large changes in the background noise level, in this case caused by traffic noise.**
(WAV)

File S3 **This file was judged by the algorithm to be analyzable (i.e., variations in the background noise level were below the threshold level) but contains a variety of potentially confusing noises.** Unlike audio file S2, this file contained several regions of fairly stable background noise which allows the noise floor to be estimated.
(WAV)

File S4 **This file was rejected because the maximum signal level was quite low, leading to poor SNR.** The low recording levels represent a technical problem in recording.
(WAV)
